# A prospective randomized controlled clinical trial of Pingmu Decoction combined with acupuncture in the treatment of non-active thyroid-related ophthalmopathy

**DOI:** 10.1097/MD.0000000000023734

**Published:** 2020-12-18

**Authors:** Hongyan Li, Pengfei Zheng, Jie Min, Yali Zhang, Wei Wang, Jingwen Zhang, Hong Li

**Affiliations:** a Department of Endocrinology, Longhua Hospital, Shanghai University of Chinese Medicine, Shanghai; b Department of Internal Medicine, 949 Hospital of PLA Army, Altay; c Stomach and Spleen Institute of Shanghai University of TCM, Shanghai, China.

**Keywords:** Graves eye disease, non-active, Pingmu Decoction, acupuncture treatment, randomized comparison clinical trial

## Abstract

**Background:**

Thyroid eye disease of orbital disease first, it is main and clinical expression with exophthalmos, visual impairment, serious influence patient's quality of life and beautiful, especially for the non-active Graves’ ophthalmopathy, there is no effective treatment methods at home and abroad, for the non-active thyroid eye disease only recommended surgery, but the procedure pain, poor curative effect, postoperative recurrence, most patients are difficult to accept. Pingmu Decoction can effectively reduce the degree of exophthalmus and TCM syndrome integral, and The combination therapy with acupuncture and moxibustion on the basis of Pingmu Decoction has achieved remarkable clinical effect, but the lack of rigorous evidence of evidence-based medicine (ebm). The test is designed to further evaluate flat mesh in active soup combined with acupuncture treatment of thyroid related ophthalmopathy card belongs to the yang qi-deficiency, phlegm and blood stasis block syndrome in patients with clinical efficacy and safety.

**Methods/Design:**

A prospective, randomized controlled clinical trial will be conducted to evaluate the efficacy and safety of Pingmu Decoction combined with acupuncture in the treatment of non-active thyroid-related eye disease in patients with Yang qi deficiency and phlegm-blood stasis syndrome. A total of 120 patients with non-active thyroid-related eye disease, namely deficiency of Yang qi and blockage of phlegm and blood stasis, are randomly divided into 3 groups and treated for 12 weeks. All three groups will maintain basic western medicine treatment. The primary outcomes are to observe the degree of prominence of eyes, and the TCM syndrome scores. The secondary result is clinical efficacy. Free triiodothyronine (FT3), free thyroxine (FT4), thyroid stimulating hormone (TSH), and thyroid stimulating hormone receptor antibody (TRAb)will be used as the observation indicators in this study. In addition, adverse reactions will be observed and recorded as safety indicators.

**Discussion:**

The results of this trial will provide convincing evidence for the efficacy and safety of Pingmu Decoction combined with acupuncture in the treatment of patients with non-active thyroid-associated eye disease with deficiency of Yang qi and phlegm and blood stasis block, and it will expand the treatment options for patients with non-active thyroid-related eye disease.

**Trial registration:**

Clinical Trials.gov ID: ChiCTR2000039626. Registered on 3 November 2020.

## 1 Introduction

Thyroid associated ophthalmopathy, also known as Graves’ ophthalmopathy (GO), is a specific autoimmune disease related to thyroid disease and involving the eye socket. Its incidence ranks the first among adult orbital diseases.^[1]^ Its pathogenesis is still unclear, and it is related to environmental, immunological and genetic factors.^[2]^ The active phase of the disease is characterized by lymphocyte infiltration, fibroblast activation and edema.^[3]^ In the non-active stage, the main clinical manifestations were orbital fibrosis, soft tissue inflammation, eye movement disorder caused by eyeball protrusion, eyelid retraction, and orbital fibroblast inflammation.^[4]^ For the treatment of thyroid-associated ophthalmopathy in the active stage, the guidelines recommend that hormone shock therapy, immunosuppressive agents, orbital radiation therapy and surgery are often used in the active stage, while targeted surgery is used to relieve ocular symptoms in the inactive stage.^[5]^

Non-active thyroid associated ophthalmopathy is a refractory disease, which seriously affects the quality of life of patients. Currently, treatment means for non-active thyroid-related eye diseases are limited, and the curative effect is poor. Therefore, a treatment method with accurate curative effect and easy to be accepted by patients is urgently needed. In recent years, it has been found that traditional medicine has unique advantages in the treatment of this disease, which can significantly reduce local inflammation, improve visual acuity and reduce the protrusion degree and the TCM syndrome score, prevent the recurrence of the disease, and reduce the adverse reactions of Western medicine. The Pingmu Decoction is a Chinese medicine consisting of Radix Astragali(Huangqi), Herba Epimedii(Yinyanghuo), Miltiorrhiza (Danshen), Sinapis Alba (Baijiezi), Oldenlandia Diffusa (Baihuasheshecao), Semen Plantaginis (Cheqianzi), etc. It has beneficial temperature Yang, dispel phlegm stasis effect, and can effectively reduce the eyeball protrusion and TCM syndrome score in patients with non-active thyroid-related eye disease, which is characterized by deficiency of Yang qi and blockage of phlegm and blood stasis.^[6]^ According to Professor Li Hong,^[7]^ Pingmu Decoction can inhibit the accumulation of post-ocular adipose tissue in GO mice by inhibiting the proliferation activity of preadipocytes, reducing the differentiation of preadipocytes into adipocytes, and promoting the apoptosis of mature adipocytes after differentiation.^[8]^ It has been found in long-term clinical practice that Pingmu Decoction combined with acupuncture has a good effect on this disease, but there is still a lack of evidence to prove it. Therefore, we designed this prospective, randomized controlled clinical trial to further evaluate the efficacy and safety of Pingmu Decoction combined with acupuncture in the treatment of non-active thyroid-related eye disease, and to provide a new treatment regimen for non-active thyroid-related eye disease.

## 2 Materials and methods

### 2.1 Study design

This is a prospective, randomized controlled clinical trial with three parallel arms. We will recruit 120 patients from The Longhua Hospital affiliated to Shanghai University of Chinese Medicine. All patients will be randomly assigned to the experimental group (Pingmu Decoction + acupuncture), control group 1 (Chinese medicine group) and control group 2 (acupuncture group). All patients will maintain basic treatment for thyroid-related eye disease: methimazole and levothyroxine sodium tablets (both from Merck & Co, LTD, Germany). Dosage will be adjusted every 28 days according to the patient's thyroid function level. Control group 1 (Chinese medicine group), Pingmu Decoction will be required to take orally twice a day, 1 bag each time., control group 2 (acupuncture group) will be treated with acupuncture alone. The experimental group was treated with pingmu Decoction combined with acupuncture. All treatments will be administered for 12 weeks.

### 2.2 Ethical issues

The trial will be conducted in accordance with the Declaration of Helsinki and Ethical Guidelines for Clinical Research, and the trial protocol has been approved by the Research Ethical Committee of Longhua Hospital Affiliated with Shanghai University of Traditional Chinese Medicine, Shanghai, China, (approval number: 2019LCSY001).

All participiants will be informed of the protocol, the purpose of the trial, and the rights, obligations, and risks involved with participation in the trial. Only patients who fully understand and sign the informed consent form will participate in the trial. In addition, the personal information about potential and e nrolled subjects will not be shared or maintained if unnecessary.

### 2.3 Study participants

We will recruit patients from the outpatient department of Longhua Hospital affiliated to Shanghai University of Chinese Medicine. The planned recruitment period is 12 months.

### 2.4 Sample size calculation

In this study, it is stipulated that only when the anterior ophthalmic process is reduced by ≥1 mm can be promoted. Exophthalmus references standard deviation is 1.3 mm, preliminary tests showed that Chinese herbal medicine could reduce the protrusion degree by 1.9 ± 1.1 mm,^[9,10]^ so we chose to reduce degree of exophthalmus to calculate the sample size, according to the α = 0.05, β = 0.1, σi is overall standard deviation for each group, μi means the population mean of each group is 1.3, K refers to the number of sample groups are 3, μ = Σμi/k, ψ of value through the check ψ of table that is 2.52, the formula for sample size = ψ2(Σσi2/k)/[Σ(μi−μ)2/(k−1)] calculated sample size is equal to about 36, considering a 10% loss to follow-up, determine the test each sample size for 40 cases, The total sample size is 120.

### 2.5 Criteria

#### 2.5.1 Inclusion criteria

Participants will be included if they fulfill the following conditions:

- Satisfy the diagnostic criteria of GO for Western medicine^[11]^ and traditional Chinese medicine, which the TCM types belong to yang-Qi deficiency and Phlegm-blood Stasis syndrome.^[12]^
- Aged 18 to 70 years
- Will be repulsing surgical treatment
- A history of GO and normal serum thyroid hormone levels (FT3, FT4) for at least 2 months
- Sign informed consent

#### 2.5.2 Exclusion criteria

Patients with any of the following conditions will be excluded:

- Failure to satisfy the inclusion criteria
- Other reasons which caused the eyeball protrusion: such as protrusion of the eye caused by nearsightedness, Primary orbital tumor, Orbital inflammatory pseudotumor, eye metastasis and other diseases
- Those with diseases of the liver, kidney, hematopoietic system, or hyperthyroidism crisis tendency and mental illness
- Those who are participating in clinical studies of other drugs
- Those who are pregnant, nursing or preparing for pregnancy

#### 2.5.3 Termination and withdrawal criteria

- Important deviations occurred in the implementation of clinical research protocols, which made it difficult to evaluate drug effects
- During the experiment, the condition of the patients continued to deteriorate and dangerous events are likely to occur
- Subjects experienced certain comorbidities, complications, or specific physiological changes during the study period
- Discontinuation of experimental drugs due to adverse events or other reasons
- Subjects do not take the test drug as required and complete the follow-up as planned, are unwilling to continue the clinical trial

#### 2.5.4 Interventions

All patients will maintain the basic treatment: methimazole and levothyroxine sodium tablets (both from Merck & Co, LTD, Germany). Dosage will be adjusted every 28 days according to the patient's thyroid function level.

Experimental group: Pingmu Decoction combined with acupuncture treatment.(Acupuncture points: Shangtianzhu, Fengchi, Sanyinjiao, Hegu, Waiguan and Yangbai are the main points, together with Cuanzhu on the head and face, Yuyao acupoints, Sizhukong acupoints, Toulinqi acupoints, Qiuhou acupoints, Qingming acupoints, Fengchi acupoints and opticoels. The index finger will be taken dajian acupoints, xiaojian acupoints wood acupoints;the pinkies will be taken Yanhuang acupoints, Huoxi acupoints;the legs will be taken Yichong acupoints. Erchong acupoints and Sanchong acupoints. Calvaria will select Baihui acupoint. All acupoints will be pierced vertically except Cuanzhu. Yuyao and Sizhukong acupoints. Frequency of acupuncture treatment: 45 minutes of needle retention twice a week). Control group 1: (Pingmu Decoction group): Patients will be given Pingmu decoction orally, 2 times a day, 1 bag each time. Control group 2 (acupuncture group): The patients will be treated only with acupuncture(acupuncture and moxibustion locations are the same as those of the experimental group). The course of treatment will be 12 weeks.

#### 2.5.5 Test the drugs and acupuncture illustrations

The drugs used in the clinical trials are Pingmu Decoction, made by The Longhua Hospital affiliated to Shanghai University of Traditional Chinese Medicine(Shanghai, China). Pingmu Decoction consists of Radix Astragali(Huangqi)15 g, Herba Epimedii(Yinyanghuo)12 g,

Miltiorrhiza(Danshen)15 g, Sinapis Alba(Baijiezi)9 g, Oldenlandia Diffusa(Baihuasheshecao)30 g, Semen Plantaginis(Cheqianzi)30 g, etc.

Decoction 200 ml/ bag, twice a day, 1 bag each time.

The acupuncture needles are provided by Suzhou Medical Supplies Factory Co, Ltd (Suzhou, China), specification: 0.25 × 25 mm. Production license number is Su Yao Food supervision apparatus production permit 20010020.

### 2.6 Randomization and allocation

We will randomize all included patients using a table of random numbers. The 120 patients will be randomly divided into the experimental group, control group 1 and control group 2 in a 1:1:1ratio according to the treatment order.

### 2.7 Outcomes

#### 2.7.1 Primary outcomes

- Exophthalmos degree. Measurement of prominence of the eyeball: Use the ocular protrusion measuring instrument (model YZ9) to measure the degree of exophthalmia(Which brand is called Crane and produced by Suzhou liuliu Vision Co., LTD.)
- TCM syndrome integral

According to the diagnostic criteria of Deficiency of Yang qi and blockage of phlegm and blood stasis formulated in 2002 edition of Clinical Research Guidelines for New Chinese Medicine.^[11]^ Main symptoms: fear of cold, lack of power, eyeball swelling pain; Secondary symptoms: tepid limbs, pale face, self - perspiration. The body of the tongue is fat or normal, the tongue is pale purple, or there are petechial spots, or the veins under the tongue are purple and angry. The tongue coating white greasy, pulse heavy fine astringent. According to the degree of occurrence of the main symptoms: no, mild, moderate and severe, they will be recorded as 0, 2, 4 and 6 points respectively, and according to the degree of occurrence of secondary symptoms: no, mild, moderate and severe, they will be recorded as 0, 1, 2 and 3 points respectively. The tongue image and pulse image of the block syndrome of phlegm and blood stasis will be scored 1 and 0 points according to whether they are consistent with deficiency of Yang qi and blockage of phlegm and blood stasis. TCM syndrome integrals will be recorded once before and after treatment in the three groups.

Efficacy of TCM symptoms will be determined by integral method: Efficacy index(n) = ((post-treatment score − pre-treatment score)/(pre-treatment score)) × 100%

#### 2.7.2 Secondary outcomes

Evaluation criteria of Western medicine curative effect:Obvious effects: symptoms such as eye distension, tearing, foreign body sensation, photophobia, burning pain, etc. basically disappeared; bulbous conjunctiva congestion and periorbital edema significantly subsided; symptoms of eye movement disorder and blepharoptosis basically returned to normal; protrusion degree decreased by ≥2 mm. Effective: the ocular symptoms and ocular signs are improved, and the protrusion degree is reduced by ≥1 mm, but the above indicators are not achieved. Useless: no significant improvement before and after treatment.

### 2.8 Safety assessments

Pingmu Decoction has been widely used in clinical practice, and has been found to be safe in clinical trials. Patients will be closely monitored for adverse reactions during this trial. In addition, laboratory tests will be performed at the beginning and end of the trial, including blood, urine, fecal, kidney, and liver function tests. If adverse events are reported, we will provide appropriate treatment to participants immediately.

### 2.9 Participant timeline

**Figure 1:**
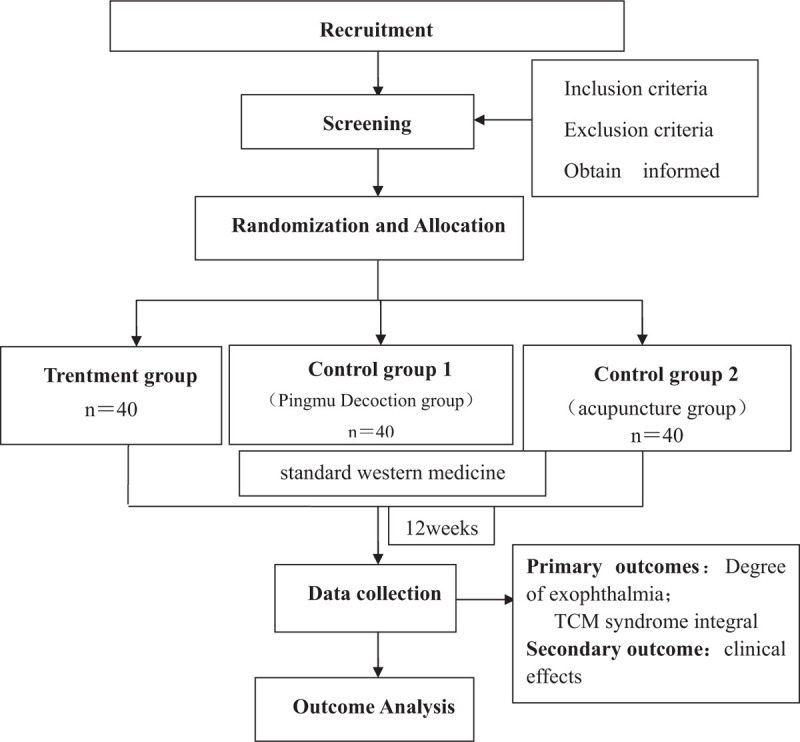
Project overview.

The study will last 12 months, from November 10, 2020 to November 10, 2021. Recruitment will start in November 10, 2020. The recruitment process is shown in Figure 1.

### 2.10 Data collection and monitoring

- Pre-clinical research training: All personnel in clinical research will be relatively fixed and receive unified training before the start of clinical trial, so that all researchers have a better understanding of the clinical trial plan.
- Data collection: Two inspectors will be appointed to oversee and inspect the research process. The data will be collected by the designated person. To ensure the accuracy of the data, two investigators will enter and proofread the data respectively. All cases will be completed in accordance with the design requirements of the Case Record Form. Withdrawal from the study and loss of follow-up (and the causes) will be fully documented.
- Quality control: The project leader supervises and checks the progress of the research, and checks whether the records and reports of the research data are true, accurate and complete. And the completed “Research Cases” for timely monitoring. All acupuncture and moxibustion operations will be carried out by specialized chief physicians to ensure the accuracy of acupuncture and moxibustion sites.

### 2.11 Statistical analysis

Statistical analysis includes statistical description and statistical inference. The statistical description of quantitative indicators includes mean, standard deviation, median, minimum and maximum. Qualitative indicators are described by frequency tables and component ratios.

All statistics will be analyzed by Statistical Packages of Social Sciences (SPSS) software (version 22.0). The continuous variables will be described using mean ± standard deviation. Continuous variables subject to normal distribution will be analyzed by Student *t* test. The Rank Sum test will be used for non-normal distribution data, ANOVA test will be used for comparison among three groups, wilcoxon test is used for non-normal distribution data. Enumeration datas will be expressed as rates, and the comparison of effective rates between groups are performed by X^2^ test. *P* < .05 will be considered statistically significant.

## 3 Discussion

Thyroid-related ophthalmopathy is a disease accompanied by symptoms such as ophthalmoptosis, diplopia, eyelid insufficiency, optic nerve injury, etc., which seriously affects the quality of life and beauty, and has the risk of blindness.^[13]^ At present, Pingmu Decoction has achieved good clinical effect in the treatment of thyroid-related eye diseases. However, the treatment of non-active thyroid-related ophthalmopathy is limited, and surgical treatment is the main treatment. but there is certain limitation of surgery, and most patients do not receive surgical treatment. Therefore, there is a lack of treatment methods that can effectively improve the patients’ eyeball protrusion, diplopia, eyelid contracture and other symptoms. At present, Pingmu Decoction has achieved good clinical effect in the treatment of thyroid-related eye diseases. Pingmu Decoction has the beneficial effect of – yang and benefiting qi, dissipating phlegm and removing blood stasis. Acupuncture can promote blood circulation around the eyes, control inflammation, and promote edema regression.^[14]^ Acupuncture can promote blood circulation around the eyes, control inflammation, and promote edema regression.^[14]^ In order to evaluate the efficacy and safety of Pingmu Decoction in combination with acupuncture, we designed this prospective, randomized, controlled trial to provide a new therapeutic regimen for the clinical treatment of non-active thyroid-related eye disease.

There exist some limitations to this study. First, this is a single-center, small scale RCT. Second, the follow-up duration is relatively short. Therefore, if we finally get a nice result from the study, we will consider conducting a multi-center, randomized, large sample and long-term clinical trials in order to provide more important informations for guiding clinical treatment.

## Acknowledgments

We would like to thank Longhua Hospital Clinical Evaluation Center, Shanghai University of Traditional Chinese Medicine.

## Author contributions

**Conceptualization:** Jingwen Zhang.

**Data curation:** Wei Wang.

**Formal analysis:** Yali Zhang.

**Methodology:** Pengfei Zheng.

**Software:** Jie Min.

**Supervision:** Hong Li.

**Supervision and guidance:** Hong Li.

**Writing – original draft:** Hongyan Li.

**Writing – review & editing:** Hongyan Li.

## References

[1] Patel A Yang H Douglas RSA New Era in the Treatment of Thyroid Eye Disease. Am J Ophthalmol 2019;208:281–8. 31377284 10.1016/j.ajo.2019.07.021

[2] Plazinska MT Sawicka-Gutaj N Czarnywojtek A Radioiodine therapy and Graves’ disease -Myths and reality. PLoS One 2020;15:1–2. 10.1371/journal.pone.0226495 PMC6957158 31929534

[3] Wiersinga WM Advances in treatment of active, moderate-to-severe Graves’ophthalmopathy. Lanc Diab Endocrinol 2017;5:134–42. 10.1016/S2213-8587(16)30046-8 27346786

[4] Eckstein A Esser J Graves’ orbitopathy. K lin Monbl ugenheilkd 2011;228:432–8. 10.1055/s-0031-1273366 21534176

[5] Bartalena L Baldeschi L Boboridis K The 2016 European Thyroid Association/European Group on Graves’Orbitopathy Guidelines for the Management of Graves’Orbitopathy. Eur Thyroid J 2016;5:9–26. 27099835 10.1159/000443828 PMC4836120

[6] Gao L Zhang X Chen W Discussion on the differentiation and treatment of Graves’ophthalmopathy in active and inactive stage. J Tianjin Univ Chin Med 2017;36:344–7.

[7] Zhang Y Wang Y Li H Influences medicated serum of Pingmu Fang and its disassembled formulas on proliferation and apoptosis of orbital preadipocytes of Graves’ophthalmopathy. J Beijing Univ Trad Chin Med 2014;3:184–9.

[8] Li H Wang Y Xu R Pingmu decoction enhances apoptosis of orbital adipocytes derived from patients with Graves“ ophthalmophathy. Mol Med Rep 2012;6:1361–6. 22972028 10.3892/mmr.2012.1080

[9] Zhang Y Wang T Li H Clinical observation on treatment of non-active graves’ophthalmopathy with syndrome of yang-qi deficiency and phlegm-blood stagnation by pingmu granule. Chinese Arch Trad Chin Med 2014;32:2124–7.

[10] Li Hong Xu Rongjuan Jianyang Clinical observation of Pingmu Decoction No. 2 in the treatment of invasive protrusion of Graves’ disease in the non-active stage. Shanghai J Trad Chin Med 2008;42:50–2.

[11] Xiaoyu Zheng Chinese medicine clinical research of new drugs guiding principles. 2002;Beijing: China Medical Science and Technology Press, 61-65.

[12] Editorial team of Chinese Society of Endocrinology, Chinese Medical Association guidelines on diagnosis and Treatment of thyroid Diseases. Chinese Guidelines for the Diagnosis and treatment of thyroid diseases 2007;46:876–82.

[13] Li H Zhang Y Zhang X Icariin Inhibits AMPK-Dependent Autophagy and Adipogenesis in Adipocytes In vitro and in a Model of Graves’ Orbitopathy In vivo. Front Physiol 2017;8:45–7. 28243204 10.3389/fphys.2017.00045 PMC5303717

[14] Ning R Acupuncture and moxibustion in the treatment of hyperthyroidism and related exophthalmia points selection rule study. Chin Acupunct Moxibust 2019;39:667–72. 10.13703/j.0255-2930.2019.06.028 31190507

